# An asexual flower of *Silene latifolia* and *Microbotryum lychnidis-dioicae* promotes sex-organ development

**DOI:** 10.1371/journal.pone.0217329

**Published:** 2019-08-16

**Authors:** Hiroki Kawamoto, Kaori Yamanaka, Ayako Koizumi, Kotaro Ishii, Yusuke Kazama, Tomoko Abe, Shigeyuki Kawano

**Affiliations:** 1 Department of Integrated Biosciences, Graduate School of Frontier Sciences, The University of Tokyo, Kashiwa, FSB, Kashiwanoha, Kashiwa, Chiba, Japan; 2 RIKEN Nishina Center, Hirosawa, Wako, Japan; University of Tsukuba, JAPAN

## Abstract

*Silene latifolia* is a dioecious flowering plant with sex chromosomes in the family Caryophyllaceae. Development of a gynoecium and stamens are suppressed in the male and female flowers of *S*. *latifolia*, respectively. *Microbotryum lychnidis-dioicae* promotes stamen development when it infects the female flower. If suppression of the stamen and gynoecium development is regulated by the same mechanism, suppression of gynoecium and stamen development is released simultaneously with the infection by *M*. *lychnidis-dioicae*. To assess this hypothesis, an asexual mutant without a gynoecium or stamen was infected with *M*. *lychnidis-dioicae*. A filament of the stamen in the infected asexual mutant was elongated at stages 11 and 12 of flower bud development as well as in the male, but the gynoecium did not form. Instead of the gynoecium, a filamentous structure was suppressed as in the male flower. Developmental suppression of the stamen was released by *M*. *lychnidis-dioicae*, but that of gynoecium development was not released. *M*. *lychnidis-dioicae* would have a function similar to stamen-promoting factor (SPF), since the elongation of the stamen that is not observed in the healthy asexual mutant was observed after stage 8 of flower bud development. An infection experiment also revealed that a deletion on the Y chromosome of the asexual mutant eliminated genes for maturation of tapetal cells because the tapetal cells did not mature in the asexual mutant infected with *M*. *lychnidis-dioicae*.

## Introduction

The basidiomycetous genus *Microbotryum* is a species-rich member of smut fungi that infect a wide range of host plants belonging to the dicotyledonous families Caryophyllaceae, Dipsacaceae, Lamiaceae, and Lentibulariaceae [1]. The smut fungus *Microbotryum lychnidis-dioicae* was isolated from *Silene latifolia* [2]. *M*. *lychnidis-dioicae* has long been used as a model for studying the ecology, genetics, and the propagation of sexual infection [3–5]. Smut disease is transmitted by smut spores attached to insects [6]. Spore germination, meiosis, and the mating of paired basidiospores of smut fungi occurs on the host cell surface [7]. The basidiospores of smut fungi form a dikaryotic mycelium after mating, which penetrates the host plant. The dikaryotic mycelium of smut fungi is observed in intercellular regions, vascular bundles, and apical meristem after invasion of the host plant. Subsequently, the dikaryotic mycelium of smut fungi forms smut spores in the pollen sacs in the blossoms of host plants [8].

*Silene latifolia* is a dioecious flowering plant with sex chromosomes in the family Caryophyllaceae; individual plants have male and female flowers. The female flowers blossom with 22 autosomes and two X chromosomes, and the male flowers blossom with 22 autosomes, one X chromosome, and one Y chromosome. *S*. *latifolia* is a model for the study of the evolution of plant sex chromosomes and ecology [9]. We require sex-chromosome-linked markers to better understand plant sex chromosomes. Therefore, several Y chromosome-linked markers were made using amplified fragment length polymorphism (AFLP) [10], random amplified polymorphic DNA (RAPD) [11], and a technique that combines laser microdissection and polymerase chain reaction (PCR) [12]. The male flowers of *S*. *latifolia* with Y chromosomes were irradiated with γ rays or heavy ion beams to produce hermaphrodites, an asexual mutant, and a pollen-defect mutant with deletions to a part of the Y chromosome [13–15]. Using the deletion status of these mutants, a map of the *S*. *latifolia* Y chromosome was created [10, 16–21]. Phenotypes differ between smut infected-male and female hosts. In the infected male, *M*. *lychnidis-dioicae* replaces the pollen in the pollen sac with its own fungal spores. In contrast, stamen formation is promoted in the infected female. Smut spores are then formed instead of the pollen in anthers [22, 23].

It is thought that three male factors are encoded on the Y chromosome of *S*. *latifolia*: gynoecium-suppression factor (GSF), stamen-promoting factor (SPF), and male-fertility factor (MFF). The asexual mutant was first isolated by Donnison et al. [14]. The asexual mutant has X and Y chromosomes, but the SPF region is deleted on the Y chromosome. The flowers of asexual mutants have a filamentous structure instead of a gynoecium in the center of the flower (as observed in males), as well as developmentally suppressed stamens at the early developmental stages [14, 24]. Sporogenous cells in developmentally suppressed anthers of the asexual mutant form in the flower buds at very early developmental stages, but parietal cell layers are absent [25].

What happens when *M*. *lychnidis-dioicae* infects asexual mutants with partial deletions in the Y chromosome? In asexual mutants, development of the stamen and gynoecium is suppressed. If suppression of stamen and gynoecium development results from the same mechanism, then suppression of gynoecium development should be released when suppression of stamen development is released by *M*. *lychnidis-dioicae* infection. In addition, what differences exist in stamens between the infected asexual mutant, the infected male, and the infected female? We infer that we are able to search for genes related to anther development, which should exist in the deleted region of the Y chromosome, by comparing the pollen sacs of stamens in the infected male, infected female, and infected asexual mutant.

In this study, progeny of the asexual mutant were successively produced by crossing the female-like flowers of the asexual mutant with the male flower of a wild-type male [24]. Five infected asexual mutants were found after inoculation with *M*. *lychnidis-dioicae* using PCR screening. We focused on gynoecium development in the infected asexual mutant and compared the successive morphological changes in floral organs caused by all *M*. *lychnidis-dioicae* infection among males, females, and asexual mutants using scanning electron microscopy and tissue section analysis.

## Materials and methods

### Plant materials and plant growth conditions

*Silene latifolia* seeds were obtained from an inbred line (K-line) and stored in our laboratory. The K-line was propagated for 17 generations of inbreeding to obtain a genetically homogeneous population. We also used asexual mutants obtained from crossing the inbred K-line with an asexual mutant (ESS1), which was originally one of the heavy-ion beam irradiation-induced Y-deletion mutants identified by Fujita et al. [20]. Plants were grown from vernalized seeds in pots in a regulated chamber at 23°C with a 16 h light/8 h dark cycle.

### *M*. *lychnidis-dioicae* inoculation

Sporidia of the *Lamole* A1 and *Lamole* A2 strains of *M*. *lychnidis-dioicae* were cultured on potato dextrose agar (BD Difco) at 23°C for 5 days and suspended at 2 × 10^6^ cells/mL in distilled water. Sporidia mixtures of A1 and A2 at equal concentrations were used throughout the inoculations. Inoculation treatments were performed on 10-day-old seedlings of *S*. *latifolia* on 0.8% agar plates. The base of each 10-day-old seedling was injected with 2 μL of the mixture. Inoculation was repeated after 3 days. Three weeks after inoculation, we transferred the seedlings to soil in pots and grew them in a regulated chamber at 23°C with a 16 h light/8 h dark cycle.

### Scanning electron microscopy

Flowers were fixed overnight in 2.5% glutaraldehyde in 0.1 M phosphate buffer (pH 7.2) at 4°C. After being washed three times with 0.1 M phosphate buffer (pH 7.2), fixed flowers were dehydrated in an ethanol series (30, 50, 70, 80, 90, 95, and 100% each step for 30 min at room temperature) and kept in 100% ethanol overnight at 4°C. The ethanol was replaced with isopentyl acetate and the flowers were dried with a critical-point dryer (HCP-2, Hitachi, Tokyo) and sputter-coated with platinum palladium using an ion sputter (E-1010, Hitachi). The flowers were examined in an S-3000N scanning electron microscope (SEM) (Hitachi) operated at 5 kV in high-vacuum mode. The gynoecium on the scanning microphotographs was colored using Photoshop 7.0 (Adobe Systems, San Jose, CA).

### Light microscopy

Flowers were double fixed overnight in 4% glutaraldehyde in 0.1 M phosphate buffer (pH 7.2) at 4°C and post-fixed for 4 h in 2% osmium tetroxide in distilled water. After being washed with 0.1 M phosphate buffer (pH 7.2), the fixed flowers were dehydrated in an ethanol series (30, 50, 70, 80, 90, 95, and 100% each step for 15 min at room temperature) and kept in 100% ethanol overnight at 4°C. The ethanol was replaced with xylene and embedded in paraffin. Embedded flowers in paraffin were cut into 10-μm sections using a microtome (RV-240, Yamato, Japan). The cut sections were deparaffinized in xylene and rehydrated in an ethanol series (100, 95, 90, 80, 70, 50, and 30%; each step for 10 min at room temperature). The rehydrated sections were stained with Schiff’s reagent. The sections were observed with a microscope (BX60, Olympus, Tokyo, Japan).

### Screening for asexual mutants with a partial Y chromosome deletion

Artificial smut infection has been established only at the cotyledon stage. Therefore, it is necessary to cross a WT male and female-like flowers of the asexual mutant to obtain progeny. The progeny are treated immediately after germination for smut infection and examined for smut spores in anthers at the first flower. To screen for asexual mutants from crossing of a sexual mutant (ESS1) and inbred line (K-line), we performed polymerase chain reaction (PCR) screening using four STS makers (MS4, MK17, ScQ14, and SlAP3) and flower phenotype screening. The STS markers (MS4, MK17, ScQ14, and SlAP3) are described in Kazama et al. [21]. Genomic DNA was extracted from fresh leaves using a DNeasy Plant Mini Kit (Qiagen), according to the manufacturer’s instructions. PCR amplification was performed using Blend Taq polymerase (Toyobo, Tokyo, Japan) with a Thermal Cycler Dice TP600 (TaKaRa Bio, Otsu, Japan). The conditions were 5 min at 94°C, followed by 35 cycles of 1 min at 94°C, 1 min at 60°C, and 1 min at 72°C, with final 5 min extension at 72°C. Each 50-μL reaction mixture contained 50 ng of template DNA, 5 μL of 2 mM dNTPs, 5 μL of 10× Blend Taq Buffer, 1 μL of Blend Taq, and 1 μL of each primer at 10 μM. The reaction products were electrophoresed on 1.5% agarose gels. After staining with ethidium bromide, fragments were visualized on a UV illuminator (Atto, Tokyo, Japan). Wild-type male and female genomic DNA was used as a control.

## Results

### Stages of flower bud development in an asexual mutant of *S*. *latifolia*

**Fig 1:**
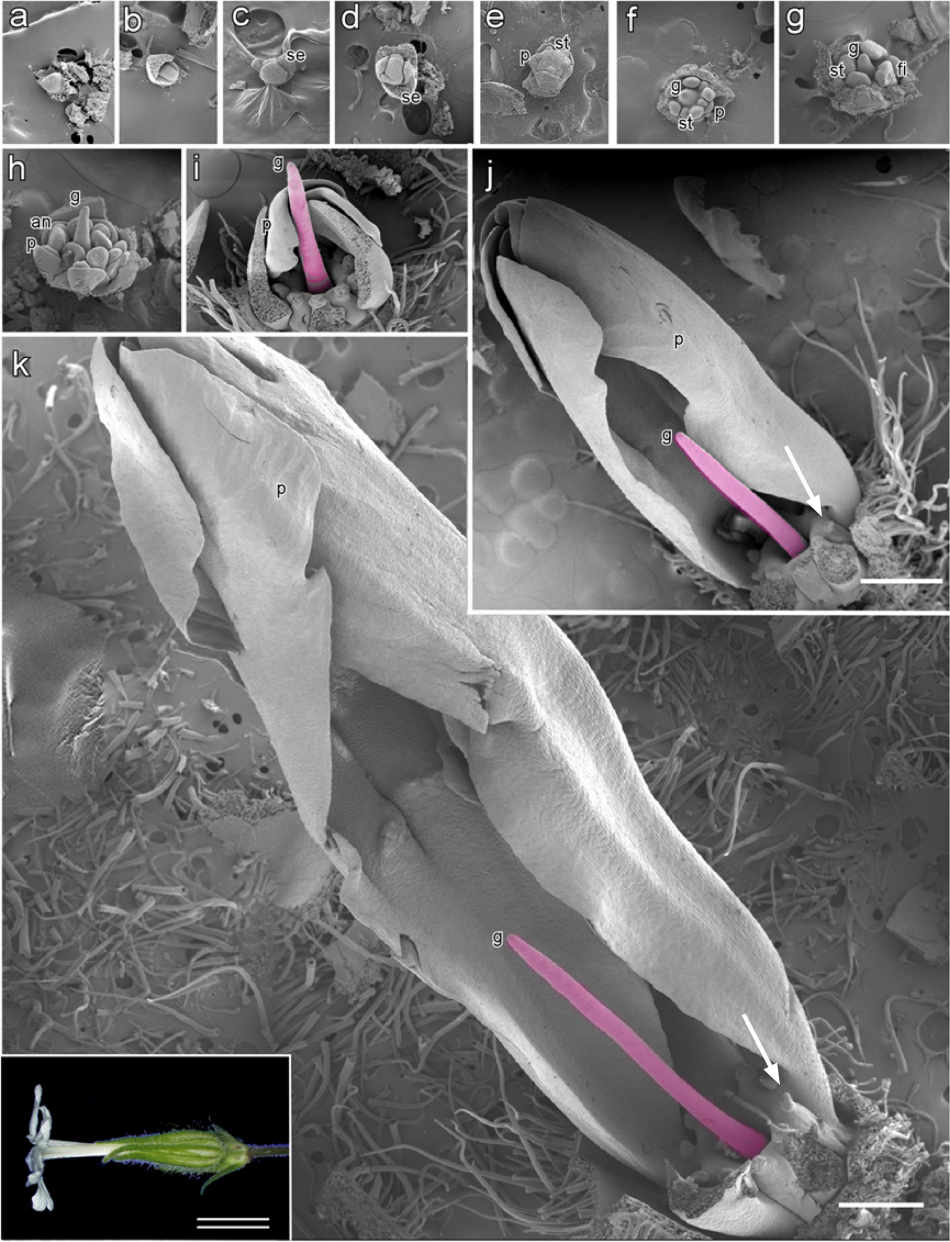
Scanning electron microphotographs of flower bud development in an asexual mutant of *S*. *latifolia*. Partially dissected asexual flower buds revealing the internal structures at successive stages of development. This study identified 11 developmental stages of the asexual flowers **(a–k)**. Stages 1 to 7 are shown in transverse view; the remaining stages are shown in longitudinal view. Early stages are observed to be bisexual **(a–f)** but later stages are not observed in gynoecium or anther development **(g–k)**. Asexual flowers at stages **a)** 1, **b**) 2, **c**) 3, **d)** 4, **e**) 5, **f**) 6, **g**) 7, **h**) 8, **i**) 9, **j**) 10, and **k**) 11. Inset: bright-field microphotograph showing an open flower. Anthers **(an)**, sepal **(se)**, stamen **(st)**, gynoecium **(g)**, petal **(p)**, and filaments **(f).** Arrows in j and k indicate suppressed anthers. Bar = 500 μm, double bar = 1 cm.

In this study, we newly defined stages of flower bud development of the asexual mutant to correspond with stages of flower bud development in male and female flowers. Stages of flower bud development of the asexual mutant were divided into 11 stages (Fig 1A–1K). Flower bud development in the asexual mutant at stages 1 to 6 was similar to that of males. The flowers of the asexual mutant with developing stamen primordia and gynoecium primordia at stages 1 to 5 of flower bud development showed hermaphroditic morphology (Fig 1A–1F). However, although expansion of the stamen filament occurred at stage 7 in male flowers [23] (S1 Fig), expansion of the stamen filament was not seen in the asexual mutant at stage 7, (Fig 1G). Stamen development was suppressed at stage 8 of flower bud development, and the gynoecium became a filamentous structure (Fig 1H). Only the petals and filamentous gynoecium developed at stages 9 to 11 (Fig 1I–1K). Suppressed anthers became trapezoidal or rectangular, similar to structures observed in the wild-type female (Fig 1).

### Morphological changes in the asexual mutant caused by *M*. *lychnidis-dioicae*

**Fig 2:**
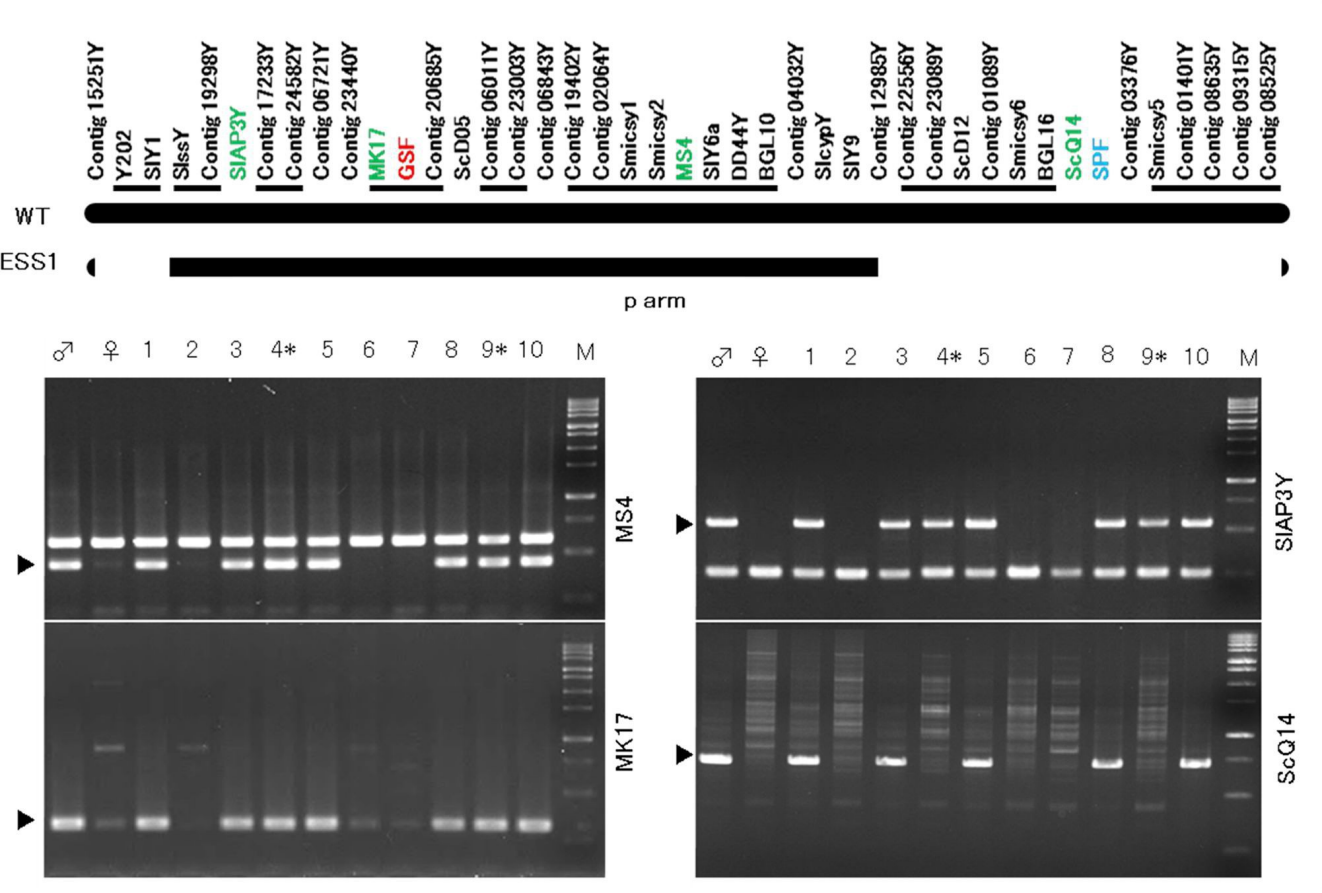
Selection of infected asexual mutants. After 188 seeds germinated, *S*. *latifolia* sprouts were inoculated with *M*. *lychnidis-dioicae*. We checked mock or infected plants after 3 months and performed genotyping by PCR using four markers: MK17, ScQ14, SlAP3, and MS4. Asterisks indicate infected asexual mutants. The black arrowhead indicates a Y-specific band. The order of the markers is as described in Kazama et al. [21]. Closely linked marker sets, in which the orders of markers are not fixed, are underlined. Numbers indicate the individual strains obtained by crossing males and asexual flower mutants. As explained above, this is the result of screening strains infected with smut fungus. *Asexual flower mutants.

Both the asexual flower and the female-like flower blossom are produced in the asexual mutant ESS1, which has one X chromosome and one Y chromosome with a deletion of the SPF region. The female flowers had five styles, but the female-like flowers had only two styles. It was possible to produce progeny of the asexual mutant by crossing the female-like flowers in the asexual mutant with the male flower in the wild-type male because gynoecia of the female-like flowers in the asexual mutant were fertile. Artificial smut infection has been established only at the cotyledon stage. Therefore, it is necessary to cross a WT male and female-like flowers of the asexual mutant to obtain progeny. The progeny are treated immediately after germination for smut infection and examined for smut spores in anthers at the first flowering. As a result, we obtained 234 seeds, and those seeds were sowed. Of the germinated seeds, 188 *S*. *latifolia* sprouts were inoculated with *M*. *lychnidis-dioicae*. We genotyped mock or infected plants 3 months after infection, based on genotyping by PCR using four markers: MK17, ScQ14, SlAP3, and MS4 (Fig 2). This produced a female with two X chromosomes, a male with a Y chromosome, and asexual flower mutants with a Y chromosome containing the deleted region and an X chromosome. However, none of the individuals without an X chromosome grew to the flowering stage. Therefore, the seed likely suffered lethality before germination.

**Fig 3:**
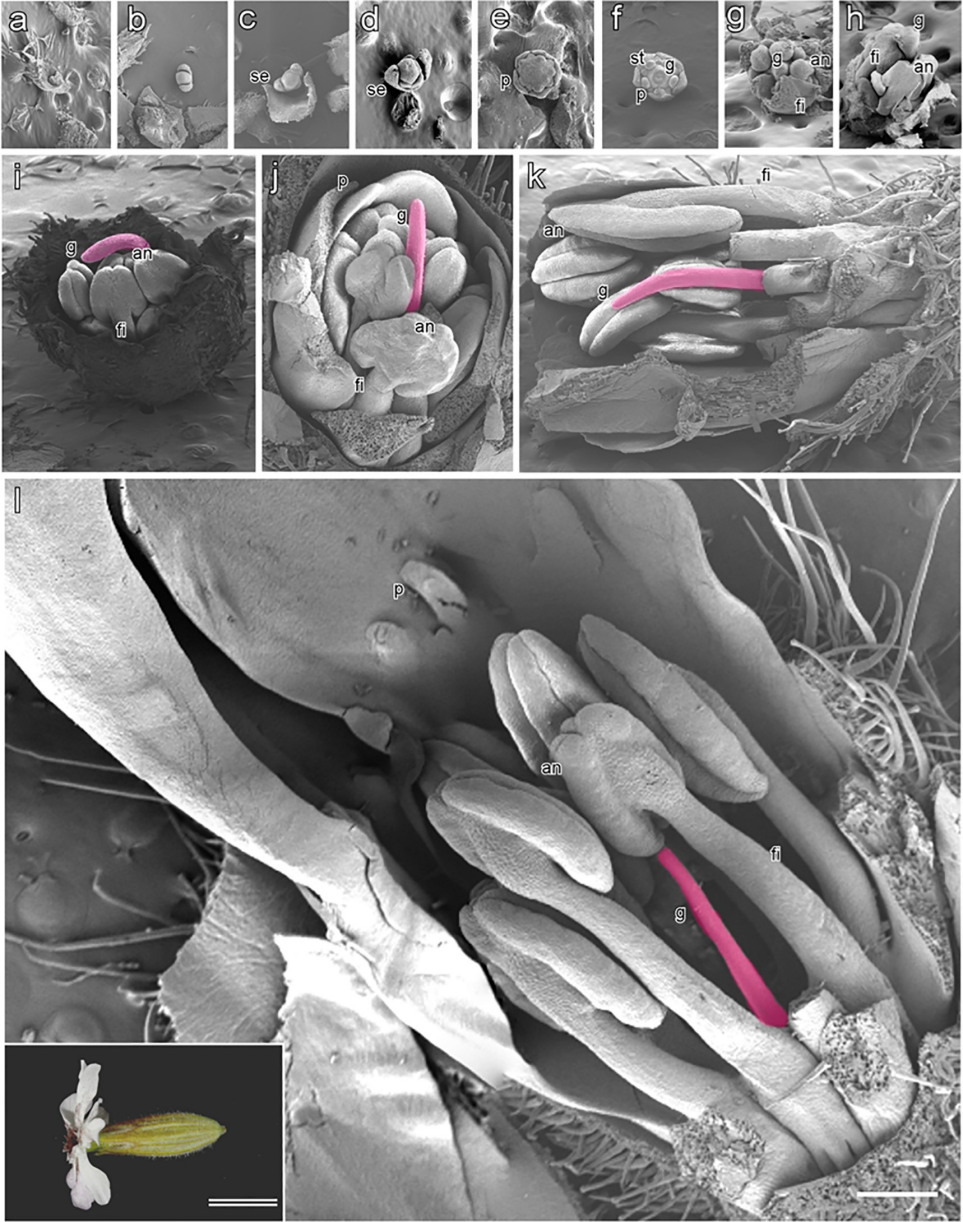
Scanning electron photomicrographs of flower bud development in infected asexual *S*. *latifolia*. Infected partially dissected asexual flower buds showing internal structures at successive stages of development. This study identified 12 developmental stages of infected asexual flowers **(a–l)**. Stages 1 to 7 are shown in transverse view; the remaining stages are shown in longitudinal view. Early stages are observed to be bisexual **(a–f)**, and later stages show anther development, which does not occur in asexual mutants **(g–l)**. Infected asexual flowers at stages **a**) 1, **b**) 2, **c**) 3, **d**) 4, **e**) 5, **f**) 6, **g**) 7, **h**) 8, **i**) 9, **j**) 10, **k**) 11, and **l**) 12. Inset: bright-field microphotograph showing an open flower. Anthers **(an)**, sepal **(se)**, stamen **(st)**, gynoecium **(g)**, petal **(p)**, and filaments **(f)**; Bar = 500 μm, double bar = 1 cm.

Table 1 shows the obtained progeny and the *M*. *lychnidis-dioicae* infection rate. We obtained 66 female individuals with two X chromosomes, 91 male individuals with the X chromosome and the intact Y chromosome, and 15 asexual mutant individuals with the X chromosome and the Y chromosome with deletion of the SPF region (Table 1). In these plants, the infected female was found as 48 individuals, the infected male was found as 54 individuals, and the infected asexual mutant was found as 5 individuals (Table 1). Flower bud development in the infected asexual mutant was divided into 12 stages (Fig 3A–3L). The morphology of the infected asexual mutant appeared to be similar to that of the male and healthy asexual mutant at stages 1 to 6 (Fig 3A–3F). However, extension of the stamen filament in the infected asexual mutant was confirmed, as well as in the male, at stages 7 and 8 of flower bud development, and developmental suppression of the stamens did not occur at stage 8 in the asexual mutant (Fig 3G and 3H).

**Table 1:** Sexual genotype and *M*. *lychnidis-dioicae* infection ratio in progeny of the cross between an asexual mutant (XY^d^) and a wild-type male (XY) of *S*. *latifolia*.

		Result of inoculations		
Phenotypes	Genotypes	Mock	Infected	Total
Female	XX	35	48	83
Male	XY	36	54	90
Asexual	XY^d^	10	5	15
Lethal	YY^d^	0	0	0
Total		71	107	188

Y^d^ indicates a Y chromosome with a deletion.

### Morphological changes of anther locule caused by *M*. *lychnidis-dioicae*

**Fig 4:**
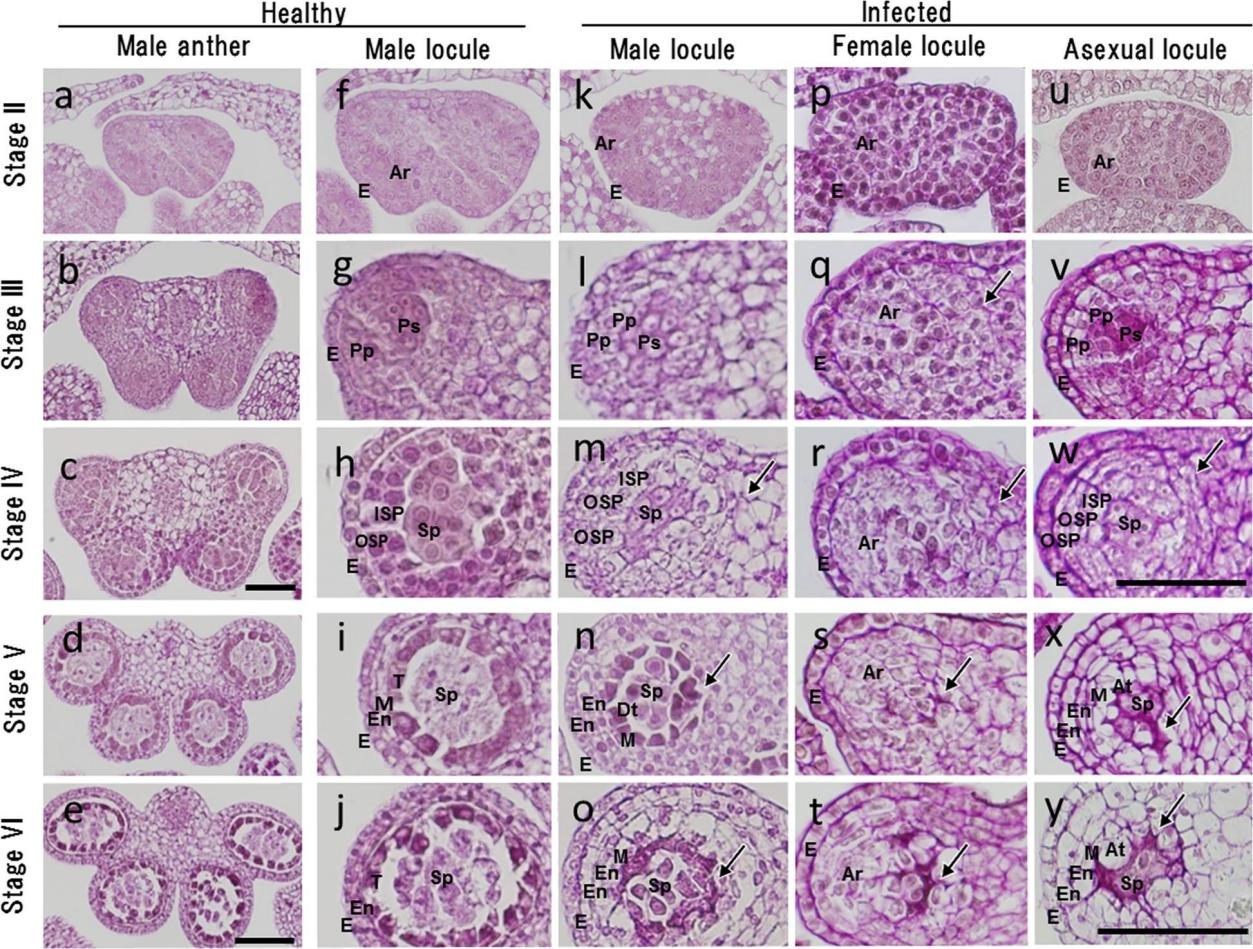
Light photomicrographs showing anther development at stages II to VI in a healthy male flower, infected male flower, infected female flower, and infected asexual flower. This study identified 14 developmental stages of healthy male flowers. Four stages of anther development in healthy male flowers and the corresponding stages of development in infected male flowers, infected female flowers, and infected asexual flowers were compared with respect to locule tissues by periodic acid-Schiff staining. The images are of cross-sections through an anther locule. In the asexual flower mutant, the stamens did not develop beyond stage III, so tapetal cells did not form. **a–e** Anther tissues of healthy male flowers at stages II to VI; **f–j** single locule of healthy male flowers at stages II to VI; **k–o** single locule of infected male flowers at stages II to VI; **p–t** single locule of infected female flowers at stages II to VI;, **u–y** single locule of infected asexual flowers at stages II to VI. Arrows indicate smut fungi. Archesporial (Ar), abnormal tapetum (At), different morphology tapetum (Dt), epidermal cells (E), endothecium (En), inner secondary parietal cells (ISP), middle layer (M), outer secondary parietal cells (OSP), primary parietal cells (PP), primary sporogenous cells (Ps), sporogenous cells (Sp), tapetum (T). **a**, **e**, **i**, **m**, **o**, bars = 50 μm; **b–d**, **f–h**, **j–l**, **r–t**, bars = 100 μm.

In this study, we observed anther locules of the healthy male, the infected male, as well as those of the infected female and infected asexual mutants (Figs 3 and 4A–4Y, and S1 Fig). Each of the infected plants was observed and compared at stages II to VI of anther development. Archesporial cells and epidermal cells formed at stage II of anther development in the healthy male (Fig 4A). The anther locule at stage V of anther development was composed of a layer of epidermis, a layer of endothecium, a middle layer, a tapetum layer and pollen mother cells as a result of the differentiation of archesporial cells (Fig 4A–4D and 4F–4I). The middle layer and the tapetum layer caused programmed cell death at stage VI of anther development (Fig 4E and 4J).

In the infected males, the primary parietal cell layer was divided into two layers at stage III of anther development (Fig 4L). Furthermore, the secondary parietal cell layer was divided into two layers at stage IV of the anther development (Fig 4M). In the infected males, the endothecium was present in the two layers, unlike in the healthy males. Furthermore, the tapetum and the middle layer in infected males showed different morphology from those in the healthy males at stage V of anther development (Fig 4N). Unlike in the healthy male, in the infected male, the tapetum rapidly disintegrated at stage VI of anther development, where the hyphae of *M*. *lychnidis-dioicae* normally grew (Fig 4O).

**Fig 5:**
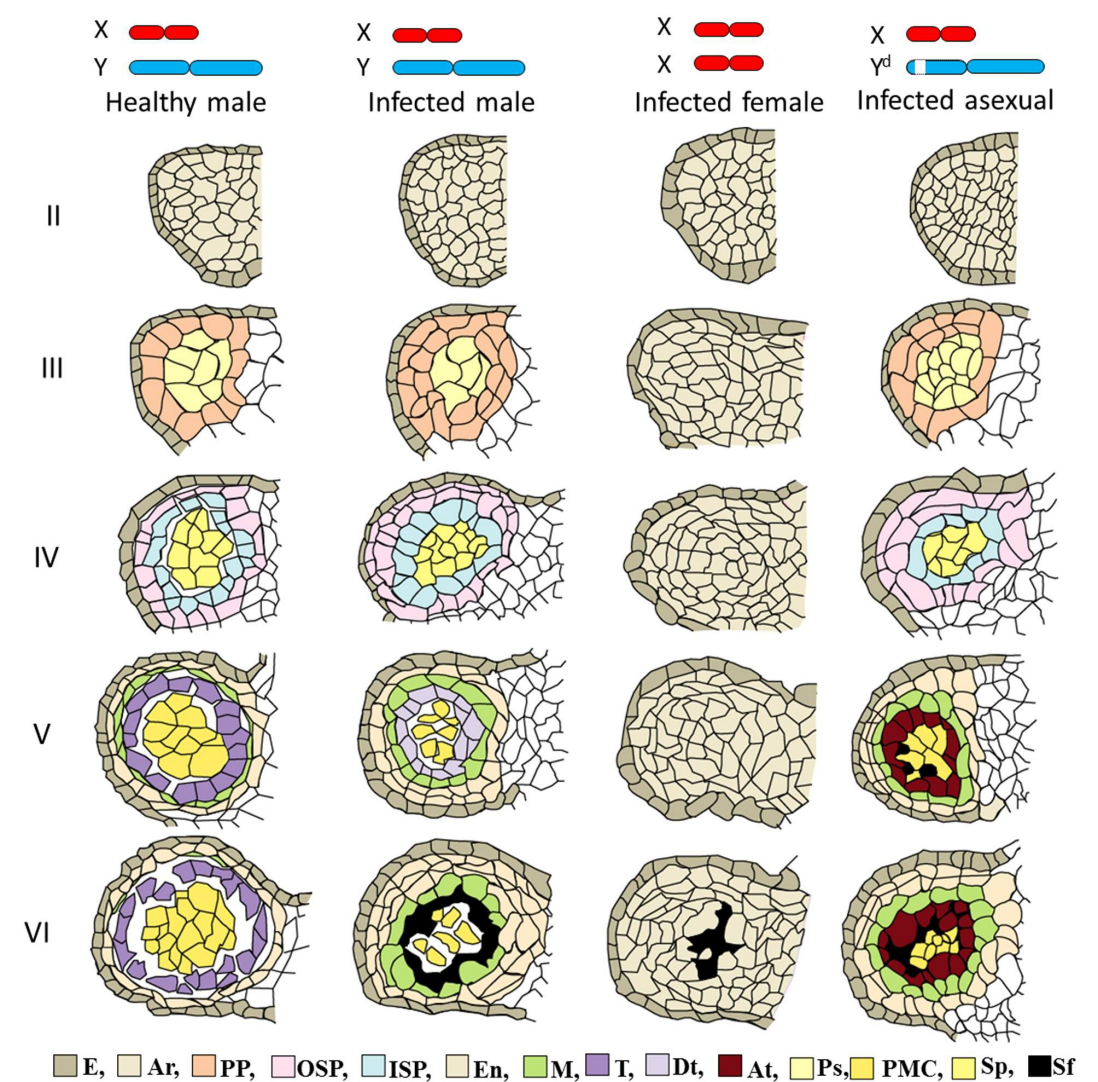
Schematic diagram of anther development in healthy male flowers, infected male flowers, infected female flowers, and infected asexual flowers. Tapetum and sporogenous tissues are seen in healthy and infected male flowers but are absent in infected female flowers. Sporogenous tissues are present only in infected asexual flowers. The different-morphology tapetum (DT) in infected male flowers, as in normal male flowers, is stained with PAS, but the abnormal-morphology tapetum (AT) in the asexual flower mutants is not. This phenomenon suggests that the AT is not maturing normally in the asexual flower mutants. In the asexual flower mutants, the stamens do not develop beyond stage III, so tapetal cells do not form. Archesporial cells (Ar), abnormal tapetum (At), different morphology tapetum (Dt), epidermal cells (E), endothecium cells (En), inner secondary parietal cells (ISP), middle layer (M), outer secondary parietal cells (OSP), pollen mother cells (PMC), primary parietal cells (PP), primary sporogenous cells (Ps), sporogenous cells (Sp), smut fungus (Sf), tapetum (T).

In the infected female, only archesporial-like-cells and epidermal cells existed, but primary parietal cells and primary sporogenous cells did not exist in stage III of anther development (Fig 4Q). Differentiation of the archesporial-like-cells did not occur at later stages (Fig 4P–4S). Growth of *M*. *lychnidis-dioicae* was also observed until stage VI of anther development in the infected female (Fig 4T). In the infected asexual mutant, development of the anther locules in developed stamens caused by *M*. *lychnidis-dioicae* was similar to that in the infected male at stages II to IV of anther development (Fig 4U–4W), whereas the morphology of the tapetum at stage V of anther development was different from that in the infected male (Fig 4X). Growth of *M*. *lychnidis-dioicae* was also observed at stage VI of anther development (Fig 4Y). Thus, it was found that *M*. *lychnidis-dioicae* in the infected asexual mutant and infected female grew at the center of the anther locule, where the tapetum was absent, although *M*. *lychnidis-dioicae* in the infected male grew along the tapetum (Figs 4N, 4S, 4X and 5).

## Discussion

### *M*. *lychnidis-dioicae* effects a change in anther developmental stages

Schematic diagrams of the developmental stage of the anther locule are shown in Fig 5 and S2 Fig Upon infection of the male flower of *S*. *latifolia* with *M*. *lychnidis-dioicae*, the endothecium was multi-layered, and the tapetum and middle layer showed different morphology from that of the healthy male. However, the endothecium, middle layer, tapetum, and pollen mother cell in the anther locule of the infected male were present as in the healthy male. The stamen of the female of *S*. *latifolia* is elongated by infection with *M*. *lychnidis-dioicae*. The anther of the female has only archesporial cells and epidermal cells in its locules and does not cause differentiation of the archesporial cells during the development of sporogenous cells at very early developmental stages. Therefore, we suggest that genes related to formation of the endothecium, middle layer, and tapetum are located on the Y chromosome.

Farbos et al. [25] observed developmentally suppressed anthers of a healthy asexual mutant at early stages of anther development. As a result, suppressed anthers of the asexual mutant formed archesporial cells, but parietal cells were absent. In this study, normal differentiation of the tapetum was not observed in the infected asexual mutant. Morphology of the anther locule in the infected asexual mutant resembled the *Arabidopsis dysfunctional tapetum1* (*dyt1*) mutant, in which the *DYT1* gene (related to differentiation of the tapetum cells) was mutated [26]. Such genes may be lacking in the asexual mutant infected with *M*. *lychnidis-dioicae*. Although ESS1 and previously identified asexual mutants [25] were produced in different ways and the extent of the contribution of the *M*. *lychnidis-dioicae* infection to the production of abnormal tapetum cells is largely unknown, it is possible that the genes related to differentiation of the tapetum and middle layer were located around the SPF region of the ESS1 Y chromosome.

### Morphological changes induced by *M*. *lychnidis-dioicae*

It is thought that elongation of the stamen occurred in the infected female of *S*. *latifolia* because *M*. *lychnidis-dioicae* plays an alternative role in the Y chromosome of infected females of *S*. *latifolia*, which lack the Y chromosome [27]. *SLM2* is a homolog of the *PISTALA* (*PI*) B-class gene and is expressed in the stamen and petal primordia [28]. It is thought that smut fungus regulates the expression of *SLM2* since this gene is not expressed in the stamen primordia of the female but expressed in the stamen primordia in the infected female [21]. In addition, *SLM2* is not expressed in the stamen primordia of asexual mutants, in which the anther development does not occur as in the female [15]. The asexual mutant has a pair of X and Y chromosomes. In the mutant, genes related to gynoecium suppression located in the GSF region are intact, whereas the SPF region is deleted. Therefore, it is thought that development of the stamen does not occur after stage 7 of flower development. This is due to the suppression of gynoecium development caused by GSF function and a lack of promotion of stamen development due to the deletion of the SPF region. Development of the gynoecium is suppressed and replaced by the filamentous structure because the GSF on the Y chromosome is intact (Figs 1 and 2). When *M*. *lychnidis-dioicae* infects the asexual mutant, the gynoecium displays a filamentous structure as in the healthy asexual flower mutant. Therefore, it is suggested that the suppression of gynoecium development was not released by the infection of *M*. *lychnidis-dioicae*. In addition, it is thought that *M*. *lychnidis-dioicae* has a function similar to SPF since the elongation of the stamen that is not observed in the healthy asexual mutant was observed after stage 8 of flower bud development.

Stamens develop only after infection with *M*. *lychnidis-dioicae*, but the maturation of tapetal cells is abnormal in the anther locule. This phenomenon suggests that the asexual flower mutants lack genes for stamen development and for tapetal cell development. In addition, infected female flowers reportedly suppress cell death and promote cell-cycle arrest in the stamen [29]. Therefore, *M*. *lychnidis-dioicae* plays a role in promoting the development of stamens with a defective Y chromosome even in asexual flower mutants. In contrast, *M*. *lychnidis-dioicae* is likely involved in the promotion of stamen development but not in its maturation because the female flower does not develop tapetal cells. The genetic map in Kazama et al. [30] suggested that the asexual flower mutant (ESS1) in this study has the smallest deletion region among the known mutants. Because the tapetal cells are not mature in the asexual flower mutant infected with *M*. *lychnidis-dioicae* (Fig 5) and based on the results of Kazama et al. [30], we speculate that the genes involved in tapetum maturation are in the vicinity of the SPF.

### *M*. *lychnidis-dioicae* localization and timing of infection influence anther locules

*Microbotryum lychnidis-dioicae* was observed among vascular bundles, intercellular regions of epidermal cells, root tip cells, and apical cells after infection [8], [31]. As a result, it is thought that *M*. *lychnidis-dioicae* penetrated into the anther through the vascular bundles, and that the timing of *M*. *lychnidis-dioicae* infection was at stage 7 of flower bud development. This is because *M*. *lychnidis-dioicae* was observed in connective cells at stage III of anther development, which corresponds to stage 7 of flower bud development [31], and development of the stamen did not occur at stage 8 of flower bud development (Fig 3). The growth area of *M*. *lychnidis-dioicae* in the anther locule is different among the infected male, the infected asexual mutant, and the infected female (Fig 4N, 4S and 4X). In the infected male, tapetal cells and pollen mother cells collapse rapidly, and *M*. *lychnidis-dioicae* hyphae grow normally [23], [31] (Fig 4O). *M*. *lychnidis-dioicae* hyphae invade the anther after its formation rather than growing rapidly [31]. We suggest that *M*. *lychnidis-dioicae* was able to recognize the tapetum, such that the growth of *M*. *lychnidis-dioicae* started with tapetum dissolution and was observed at the position where the tapetum cells were present (Fig 4X). Alternatively, *M*. *lychnidis-dioicae* is reported to grow around tapetal cells because these are thinner and more fragile than other cell types [31]. In the absence of, or in the presence of immature, tapetal cells, the lack of disintegrating cells made *M*. *lychnidis-dioicae* appear to grow in the middle of the anther. Therefore, we suggest that the different growth areas of *M*. *lychnidis-dioicae* observed in infected males, infected asexual mutants and infected females was due to the absence of, or the presence of an immature, tapetum.

## Supporting information

S1 Fig Scanning electron microphotographs of female flower bud development in infected *S*. *latifolia*. Infected partially dissected female flower buds revealing internal structures at successive stages of development. This study identified 12 developmental stages of infected asexual flowers (**a-n**). Stages 1 to 7 are shown in the transverse view; the remaining stages are shown in the longitudinal view. Early stages are bisexual (**a-f**) and later stages show pistil development (g-l). Infected female flowers at stages **a**) 1, **b**) 2, **c**) 3, **d**) 4, **e**) 5, **f**) 6, **g**) 7, **h**) 8, **i**) 9 [anthers], **j**) 10 [anthers], **k**) 11, and **l**) 12. Inset: bright-field microphotograph showing an open flower. Anthers (an), sepal (se), stamen (st), Style (sty), gynoecium (g), petal (p), and filaments (f), Bar = 500 μm, Double-bar = 1 cm.
(TIF)

S2 Fig Schematic diagram of anther cell fate in healthy male flowers, infected male flowers, infected female flowers, and infected asexual flowers. Archesporial cells (Ar), abnormal tapetum (At), different morphology tapetum (Dt), epidermal cells (E), endothecium cells (En), inner secondary parietal cells (ISP), middle layer (M), microspore (Ms), outer secondary parietal cells (OSP), pollen mother cells (PMC), primary parietal cells (PP), primary sporogenous cells (Ps), sporogenous cells (Sp), smut fungus hyphae (Sfh), tapetum (T), tetrads (Tds).
(TIF)
