# Supplementary figures and images for: An asexual flower of Silene latifolia and Microbotryum lychnidis-dioicae promotes sex-organ development
Source: PLoS One. 2019 Aug 16;14(8):e0217329. doi: 10.1371/journal.pone.0217329 (PMC6697354; doi:10.1371/journal.pone.0217329)

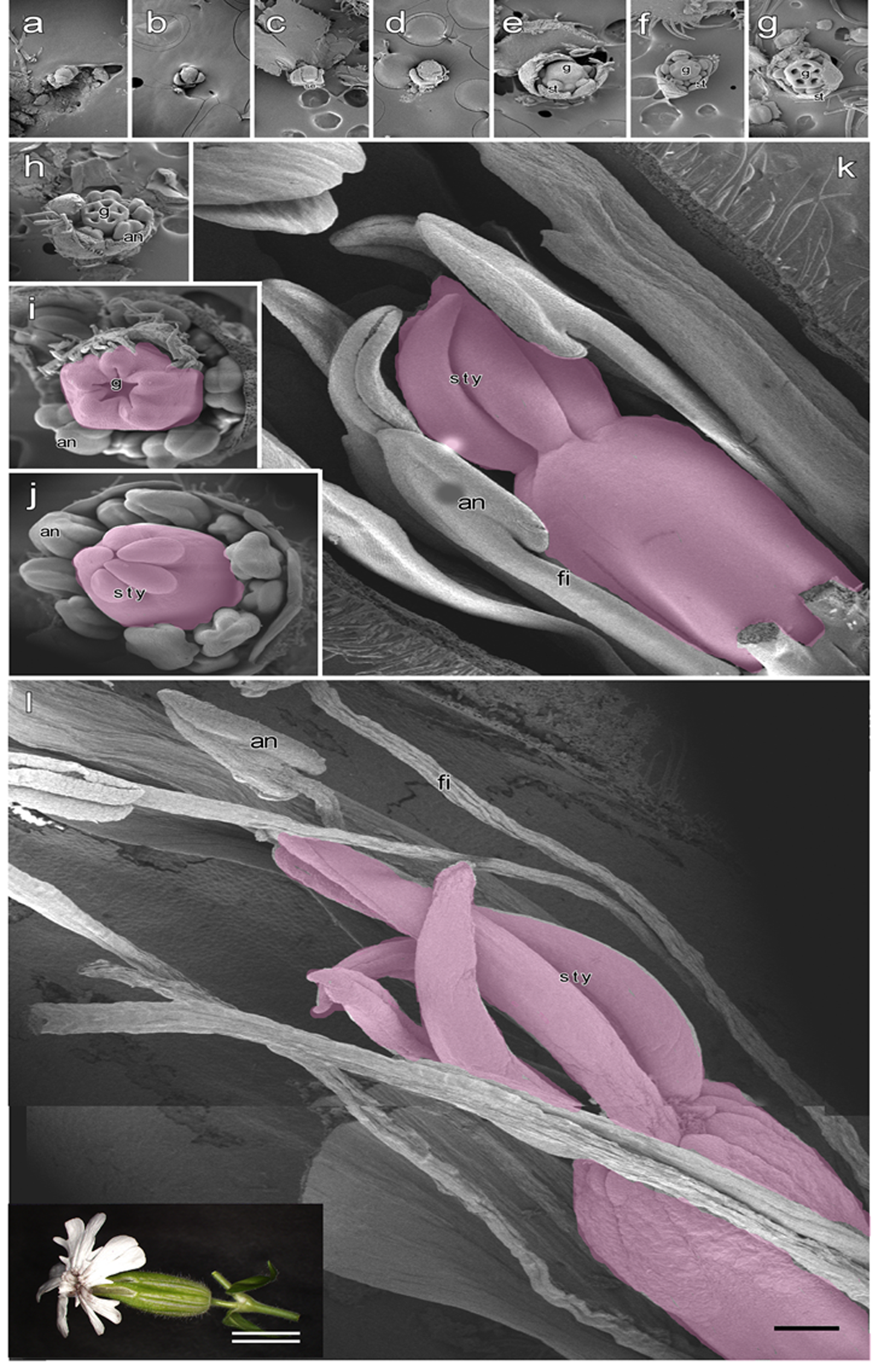

Supplement: S1 Fig — Infected partially dissected female flower buds revealing internal structures at successive stages of development. This study identified 12 developmental stages of infected asexual flowers (a-n). Stages 1 to 7 are shown in the transverse view; the remaining stages are shown in the longitudinal view. Early stages are bisexual (a-f) and later stages show pistil development (g-l). Infected female flowers at stages a) 1, b) 2, c) 3, d) 4, e) 5, f) 6, g) 7, h) 8, i) 9 [anthers], j) 10 [anthers], k) 11, and l) 12. Inset: bright-field microphotograph showing an open flower. Anthers (an), sepal (se), stamen (st), Style (sty), gynoecium (g), petal (p), and filaments (f), Bar = 500 μm, Double-bar = 1 cm. (TIF) [file pone.0217329.s001.tif]

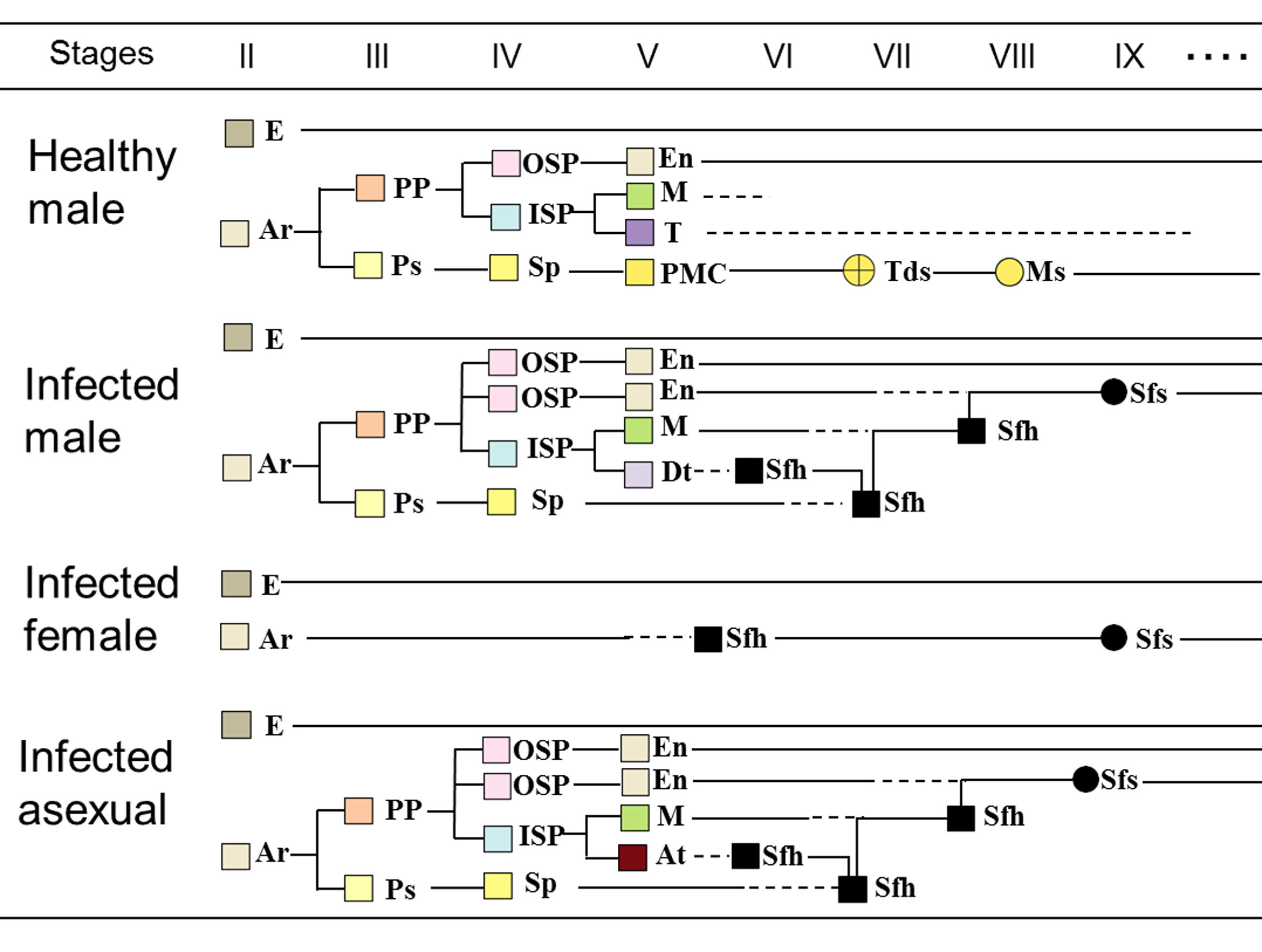

Supplement: S2 Fig — Archesporial cells (Ar), abnormal tapetum (At), different morphology tapetum (Dt), epidermal cells (E), endothecium cells (En), inner secondary parietal cells (ISP), middle layer (M), microspore (Ms), outer secondary parietal cells (OSP), pollen mother cells (PMC), primary parietal cells (PP), primary sporogenous cells (Ps), sporogenous cells (Sp), smut fungus hyphae (Sfh), tapetum (T), tetrads (Tds). (TIF) [file pone.0217329.s002.tif]
